# State Practitioner Insights Into Local Public Health Challenges and Opportunities in Obesity Prevention: a Qualitative Study

**DOI:** 10.5888/pcd11.130260

**Published:** 2014-03-13

**Authors:** Katherine A. Stamatakis, Moira Lewis, Elaine C. Khoong, Claire LaSee

**Affiliations:** Author Affiliations: Moira Lewis, Elaine C. Khoong, Washington University School of Medicine and the Prevention Research Center in St. Louis, Missouri; Claire LaSee, Washington State Department of Health, Tumwater, Washington.

## Abstract

**Introduction:**

The extent of obesity prevention activities conducted by local health departments (LHDs) varies widely. The purpose of this qualitative study was to characterize how state obesity prevention program directors perceived the role of LHDs in obesity prevention and factors that impact LHDs’ success in obesity prevention.

**Methods:**

From June 2011 through August 2011, we conducted 28 semistructured interviews with directors of federally funded obesity prevention programs at 22 state and regional health departments. Interviews were transcribed verbatim, coded, and analyzed to identify recurring themes and key quotations.

**Results:**

Main themes focused on the roles of LHDs in local policy and environmental change and on the barriers and facilitators to LHD success. The role LHDs play in obesity prevention varied across states but generally reflected governance structure (decentralized vs centralized). Barriers to local prevention efforts included competing priorities, lack of local capacity, siloed public health structures, and a lack of local engagement in policy and environmental change. Structures and processes that facilitated prevention were having state support (eg, resources, technical assistance), dedicated staff, strong communication networks, and a robust community health assessment and planning process.

**Conclusions:**

These findings provide insight into successful strategies state and local practitioners are using to implement innovative (and evidence-informed) community-based interventions. The change in the nature of obesity prevention requires a rethinking of the state–local relationship, especially in centralized states.

## Introduction

A robust public health response is needed to stem the epidemic of obesity in the United States. More than one-third of the US population is now obese (1). Geographic and culturally distinct patterns exist at the local level, underscoring the importance of a dedicated local public health effort. In 2007, county-level obesity prevalence ranged from 12% to 44%; more than two-thirds of counties in southern states had an obesity prevalence of 31% or more (2).

The extent of obesity prevention activities conducted by local health departments (LHDs) varies widely. A recent study that examined LHD prevention activities related to county-level obesity prevalence identified a gap in the LHD response to the epidemic (3). Local public health jurisdictions with high obesity prevalence were no more likely to deliver programs than those with low prevalence, suggesting that local infrastructure may be lacking where prevention is most needed. Even after obesity had become an explicit national public health priority (4), only about half of LHDs had active efforts in obesity prevention (5,6). Some data suggest that in counties where LHDs have maintained obesity prevention programs, over time obesity prevalence began to decline (7).

Many programs have invested government and foundation resources in comprehensive obesity-prevention approaches, including funding local organizations, providing policy advocacy, and strengthening evidence-based practices (8,9). Increasingly, the approach is based on a socioeconomic framework that emphasizes improving opportunities for healthy choices in communities, particularly where such opportunities are lacking (10–12). This reorientation from individual behavior-change models to policy, systems, and environmental change requires a new set of skills that mark a departure from traditional approaches to health promotion (13,14). The implementation challenge may be greater at local levels given the large degree of variability in funding structures, areas of program focus, and capacity to integrate innovations into existing practice (15).

Identification of structural factors related to LHD success in implementing innovative approaches is needed. The purpose of this qualitative study was to characterize the role of LHDs in obesity prevention and identify barriers and facilitators of successful local practice, on the basis of interviews conducted with state obesity prevention program directors.

## Methods

This qualitative study consisted of 28 semistructured interviews with state and regional health department employees from 22 states, conducted from June 2011 through August 2011. All consenting respondents were interviewed via telephone by a graduate student who was trained in interviewing techniques by experts at the local institution. Each of the 28 interviews was audio recorded and transcribed verbatim. The study was approved by the institutional review board at Washington University in St. Louis.

A series of open-ended questions was developed to elicit the breadth of obesity prevention efforts at LHDs, barriers and facilitators to prevention efforts, and ideas to improve practice (Appendix). Additional probes were created to allow further exploration of ideas that emerged during the interview. The interview guide (Appendix) was pilot-tested with current and former public health practitioners and researchers.

Participants were recruited from the 25 states receiving funding through the Centers for Disease Control and Prevention’s (CDC’s) State-Based Nutrition and Physical Activity Program to Prevent Obesity and Other Chronic Diseases. Key informants were identified from the list of program directors on the CDC website (16). Researchers contacted potential participants initially by e-mail to familiarize them with the project and then by telephone to request their participation. An additional 6 participants from 3 states were recruited through initial interviewees.

We used a general inductive approach to guide our data analysis (17). Because the interview was semistructured, a preliminary codebook with themes was created in advance by members of the research team (K.A.S., M.L., C.L., E.K.). Additional themes were identified as they emerged from the data. Two members of the research team reviewed the transcripts to ascertain whether any additional codes were needed (C.L., E.K.). This process continued until agreement was reached that the codebook contained all relevant themes. Two researchers independently coded each transcript using the finalized codebook. Inter-rater reliability from this initial round of results was assessed by calculating κ for 10 different codes on a selected portion of 6 randomly chosen interviews (mean κ = 0.47) (18). Conflicts in coding were reconciled by discussing the discrepancies and reaching a consensus (K.A.S., M.L., C.L., E.K.). Coded transcripts were then stratified into 2 groups on the basis of the state’s public health governance structure as defined by the Association of State and Territorial Health Officials (ASTHO) (19). Centralized states were defined as such if employees of local health units were considered state employees, and decentralized states were defined as such if local health units were led by employees of local governments. Two respondents represented states with mixed or shared structures, each of which was grouped with the centralized or decentralized states on the basis of the predominant similarity of described practice structures. We identified and analyzed recurring themes and key quotations for the 2 groups of coded transcripts.

## Results

The final sample included 28 key informants from 22 states, representing 6 with centralized and 16 with decentralized governance structures. The 3 main themes that emerged from the analysis were 1) role of the LHD, 2) barriers to LHD success in obesity prevention, and 3) facilitators of LHD success in obesity prevention (Table).

**Table:** Summary of Emergent Themes Related to Local Health Department Facilitators and Barriers to Obesity Prevention, Organized by State-Level Governance Structure, 2011

Characteristic	Decentralized	Centralized
Facilitators	• State support • Dedicated local health department staff • Communication networks • Community health needs assessments	• Community health needs assessments

Barriers	• Competing priorities • Lack of capacity at the local level • Siloed public health structures • Lack of local engagement in policy and environmental change	• Competing priorities • Lack of capacity at the local level • Siloed public health structures • Lack of local engagement in policy and environmental change

### Roles

In decentralized systems, participants reported that LHDs had a larger stake than did the state health department (SHD) in prioritizing local strategies. LHDs were more familiar with their communities and were thus better positioned to build coalitions to ultimately inform and change policy.

In centralized states, the LHD workforce was often seen by state practitioners as lacking the experience to take a leadership role in prevention efforts. However, there was recognition that too much involvement by the state at the local level did not always produce the desired effect. Some states were able to support LHDs as leaders by providing training and technical assistance (TA) and by acknowledging LHDs’ strategic ability to build local coalitions.

### Barriers to success

Obesity prevention coordinators, regardless of the type of state governance, agreed that LHDs did not focus enough on policy and environmental change and that a combination of structural and organizational issues stood in the way. Barriers cited included competing priorities, lack of capacity, siloed public health structures, and a lack of local engagement.

Some communities faced more immediate threats to health and well being that took priority over the less-pressing threat of obesity. Particularly in communities with socioeconomically disadvantaged populations, issues such as crime, school dropouts, teen pregnancy, and gangs took precedence. As in the community, the LHD itself was faced with prioritizing efforts relating to a range of health threats, many of which addressed more immediate conditions. In addition, the approaches that local public health officials used to address obesity prevention were often the strategies least supported by research.

Some of the barriers were the local community’s interest and focus on non-evidence-based initiatives. . . . They [the LHD] really liked doing community health fairs, but these didn’t really lead to more sustainable policy change in the community.

Another barrier to progress was the lack of capacity in LHDs. Many were so underresourced that they were unable to expand beyond their traditionally mandated and funded roles (eg, communicable disease reporting, emergency preparedness). Limited workforce skills for policy and environmental change were also mentioned.

[This is] still a very new . . . different skill set. They’re asking local health to talk to planners, municipal engineers, mayors. . . . Policy and environmental change is a new and different way of doing business.

The siloed structure of public health organizations was cited as a barrier to conducting crosscutting work. For example, the structure of categorical funding reflected programmatic siloes and hindered collaborative work by limiting efforts within the confines of outdated boundaries. In addition, the traditional structure of LHDs creates challenges to developing new partnerships across disciplines and sectors.

You have departments and divisions that are doing what they’ve always done in their own world. You’re asking people to partner who haven’t traditionally done a lot of this in the past.

When state coordinators led local efforts, particularly in centralized states, barriers were often created. There were disconnects between the state leader and local communities, resulting in communities that were often unaware of the relevance of state-identified public health priorities. The SHD’s distance from local concerns, politics, and culture and lack of contacts with local organizations also made it difficult to mobilize efforts and effect change. Local autonomy was limited, particularly in communities that did not carry out their own community health assessment (CHA). This barrier was not cited by respondents in decentralized states, where LHDs inherently have more autonomy.

We [the SHD] were the lead agency. I think we did too much instead of passing it to the community and letting them take it and run with it. This might not have given them the best tools.

### Facilitators of success

Practitioners in decentralized states were able to identify several key facilitators that bolstered LHD efforts in obesity prevention: state support, dedicated staff, a communication network, and CHAs. In contrast, with the exception of the locally implemented CHA, program coordinators in centralized states were unable to identify many facilitators to successful LHD practice.

State support, in the form of funding, TA, training, and guidance to local counterparts, was identified by state practitioners as enabling LHD success. In addition, states supplied epidemiologic data that established a need to address obesity-related outcomes and risk factors. States also served as an initial convener of coalitions, bringing together diverse community members before funding was in place. State experts from various agencies, including agriculture, commerce, and transportation, were able to help by informing initial strategic planning and continuing to serve as a resource. Identifying sources of training, educational support, and funding was essential. LHDs that received and implemented lessons from TA training were better able to take action in policy, systems, and environmental change.

Successful LHDs attended both regional and national trainings that provided them with the skills to approach this professionally. They received funding [and] state-supported TA and had success with low-hanging fruit that motivated them to continue and progress.

A pivotal facilitator was having a dedicated, full-time staff member working in obesity prevention at the local level. The prevailing view was that aggressive cutbacks and weak funding left local staff stretched to meet mandated public health activities with little time for coalition building, training, and local strategic planning. When LHDs were able to fund staff, it was often through more temporary mechanisms such as supplemental grants, which often fund only part-time positions or a portion of existing staff time.

It does take one full-time employee to really be successful. Many of our LHDs don’t have a full-time person to work on obesity prevention.

Consistent multilateral communication was another facilitator of success. Regular TA calls provided open forums across LHDs. This communication allowed them to pool problem-solving and training resources, to identify local expertise, and then to have a team approach that mobilized and synergized efforts. Through this mechanism, LHDs with greater capacity could assist those in rural counties that had more limited resources.

. . . projects that have been really successful [at] creating change relied on partnerships. The SHD was providing training opportunities at the state and local level and having regular TA calls where grantees could talk to [a] state TA advisor and each other. There were site visits. In the end we’re all better because of it.

Both centralized and decentralized states agreed that when communities carried out CHAs, obesity prevention efforts were more successful. Components of CHAs varied, with examples including thorough assessments of health outcomes, asset mapping, and guiding frameworks such as Mobilizing for Action through Planning and Partnerships. Output from the assessments included writing obesity prevention into community health improvement plans (CHIPs), forming coalitions, and specifying assets and deficits in the community. This improved local prioritization of obesity prevention and increased involvement of community leaders, foundations, and elected officials.

I think a big benefit . . . is that our . . . local public health leader[s] are the lead for all community activities including the CHA. That has really made it more successful.

## Discussion

The role that LHDs were described as playing in obesity prevention and the types of relationships that exist between state and local agencies varied across the states included in this study, with distinct patterns reflecting governance structure (decentralized vs centralized). Although several obstacles to a robust local response were noted, many structures and processes that bolstered obesity prevention efforts in LHDs were also described. These findings underscore some of the challenges to implementing obesity prevention programs and activities in LHDs and provide insight into the strategies state and local practitioners are using to successfully, and often synergistically, implement innovative (and evidence-informed) community-based interventions.

Unlike other areas of public health practice that have clear, concise sets of guidelines grounded in scientific evidence, the course is much less defined in obesity prevention. For example, many evidence-based recommendations guide specific public health actions related to tobacco-use cessation, as described in the *Guide for Community Preventive Services* (20). In obesity prevention, guidelines offer a much more heterogeneous range of recommendations related to weight reduction, physical activity promotion, and nutrition interventions, which vary widely in the strength of evidence. Other resources for recommended practices in obesity prevention based on expert consensus provide a varied list of potential activities, many geared toward policy and environmental change to increase opportunities for healthful eating and active living (14,21,22). Although these practices offer promising avenues, their successful implementation requires a high level of skill and experience as well as the cooperation of non–public-health sectors. A study conducted in California by Schwarte et al (23) found that the obesity prevention efforts of many LHDs were organized, staffed, and trained to focus on individual education rather than environmental factors, such as the availability of healthful food options. The authors identified a need for greater local workforce capacity and particularly new skills in areas of policy change, land use, and transportation, and the ability to form and mobilize partnerships across sectors and to take a community leadership role in guiding evidence-based practice.

Whether there is widespread local infrastructure to support LHD practice in obesity prevention is uncertain. Between 2008 and 2010, US LHDs lost approximately 12,000 employees (24), and primary prevention programs were prominent among those most frequently eliminated (25). LHD activity in the area of policy, thought to be the most reliable driver of health outcomes, is not strong and particularly low in rural areas (26). Furthermore, communication networks between LHDs across the nation are sparse (27). However, a focus on workforce training is linked to quality improvement and accreditation, creating opportunities to strengthen dissemination and implementation of innovative practice (28). As an example, the Public Health Accreditation Board requires agencies to conduct CHAs and CHIPs as a prerequisite to application (29). Through this process, it follows that LHDs with a high burden of obesity will prioritize obesity prevention and develop strategies for its amelioration through policy change and evidence-based practice.

Structural differences in how state and local agencies divide authority vary across states and affect the roles LHDs play in obesity prevention. More than half of US states have decentralized governance systems (representing 68% of LHDs) where local public health organizations have decision-making authority (6). In states with centralized governance, local health units are primarily led by employees of the state that retains authority over local decisions. Five states have mixed or shared authority, but pivotal decision-making power is still retained by the state (19). Although our classification for state public health governance structure was based on an established scheme by ASTHO (19), structures may be operationalized differently across programs. Therefore, it is important to interpret findings in terms of generalizable groupings while also considering the context within which public health operates in each state and locality.

Our results reflect the experiences and perceptions of state-level respondents administering a federally funded obesity prevention program in a selection of states; patterns of practice may differ substantially in states and localities not included in this sample. When this study was conducted, only half of the states received federal funding for obesity prevention; there is now funding (via CDC-RFA-DP13–1305) to all states. Also, the perspectives of practitioners on successful practice in this state-level sample may differ from those of local practitioners. Although their perspectives may allow comparisons across local models with which they have interacted, they do not represent the direct experience of local practitioners. In addition, our sample included various contexts that may encompass competing viewpoints of the role of public health in community life, which may influence the type of activities conducted in obesity prevention (especially pertaining to policy and environmental changes). Finally, local efforts in obesity prevention are not confined to LHDs. To fully characterize local public health efforts, it is important to examine how LHDs connect with others at the local level.

The change in the nature of obesity prevention requires a rethinking of the state–local relationship, especially in centralized states. For example, to the extent that obesity prevention has historically relied on various forms of nutrition education, centralized models of public health may have been more supportive. However, now that obesity prevention increasingly involves policy and environmental approaches, requiring cross-sector linkages with other community members, agencies and institutions, it requires strong local roots. The ideal system for implementing effective, evidence-informed public health interventions likely involves a combination of community linkages and leadership with a vertical information exchange. To scale up evidence-informed practices across multiple settings, a better understanding of strategies to improve public health systems and structures around obesity prevention is needed, on the basis of implementation frameworks that account for the challenges faced by LHDs (30).

## Interview Guide for State Practitioners on Local Practice Models in Obesity Prevention

1. In your state are there some examples of local communities that have made progress in obesity prevention?
  - If so what did they do?
  - What do you think contributed to this success?
    - Is there a particular organizational structure that seems to contribute to success?
    - Are there certain processes or other characteristics you can think of?
    - What does it take to do this work, to keep moving, and to be successful?
  - What role did the local health department, or LHD, play in this example?
2. Can you think of an example of a local community where obesity prevention efforts did not work out as well as you had hoped?
  - What do you think were some of the barriers?
    - Was there a particular organizational structure that seemed to pose a barrier?
    - Are there certain processes or other characteristics you can think of?
  - What role did the LHD play in this example?
3. Describe an effort within the state where the LHD played an important leadership role.
4. How have you seen LHDs communicate their ideas or get involved with advocacy or policy changes about obesity prevention?
5. In what other ways do you think the LHD could or should be involved?
6. What do you think is most conducive to being successful at the local level?
